# Awareness of the Risk Factors for Heart Attack Among the General Public in Pahang, Malaysia: A Cross-Sectional Study

**DOI:** 10.2147/RMHP.S281285

**Published:** 2020-12-23

**Authors:** Abdullah Abdulmajid Abdo Ahmed, Abdulkareem Mohammed AL-Shami, Shazia Jamshed, Mohammed Zawiah, Mohamed Hassan Elnaem, Mohamed Izham Mohamed Ibrahim

**Affiliations:** 1 Department of Pharmacy Practice, Kulliyyah of Pharmacy, International Islamic University Malaysia, Kuantan, Pahang, Malaysia; 2 Quality Use of Medicines Research Group, Kulliyyah of Pharmacy, International Islamic University Malaysia, Kuantan, Pahang, Malaysia; 3 Department of Clinical Pharmacy and Practice, Faculty of Pharmacy, UniSZA, Kuala Terengganu 22000, Malaysia; 4 Discipline of Clinical Pharmacy, School of Pharmaceutical Sciences, Universiti Sains Malaysia, Penang, Malaysia; 5 Department of Pharmacy Practice, Faculty of Clinical Pharmacy, Al Hodeidah University, Al Hudaydah, Yemen; 6 Clinical Pharmacy and Practice Department, College of Pharmacy, QU Health, Qatar University, Doha, Qatar

**Keywords:** knowledge, risk factor, cardiovascular disease, modifiable risk factors, layman, Malaysia

## Abstract

**Background:**

Cardiovascular disease is a leading nationwide cause of morbidity and mortality. Public awareness of risk factors for heart attacks is thought to impact the burden of disease, prevention, and timely management. The objective of this study was to assess the awareness of risk factors for heart attack and to identify the factors associated with the awareness of all modifiable risk factors for heart attack in the general population.

**Methods:**

This is a cross-sectional study conducted among 393 adult individuals in Kuantan, Pahang, Malaysia. Data collection was conducted through face-to-face interviews among the lay public members who were 18–64 years old, excluding healthcare professionals in clinical settings and academic settings. Statistical analysis was performed using chi-square test and logistic regression analysis.

**Results:**

The majority of the individuals identified smoking as a risk factor for heart attack, followed by atrial fibrillation (57.7%), heart disease (54.1%), and obesity (53.8%). However, diabetes (26%) was the risk factor that was least recognized by the participants. A total of 90.6% of participants identified at least one risk factor for heart attack, while 9.8% of the participants did not identify any risk factors for heart attack, whereas 5.6% identified all modifiable heart attack risk factors. Furthermore, participants aged 46–64 years old, married respondents, and Chinese participants, those with higher educational levels, and received prior information demonstrated great awareness of eight modifiable risk factors for heart attack. Multivariable logistic regression presented that participants with aged 55–64, those with family history of heart attack and individuals with dyslipidemia were factors independently related to excellent awareness (p=0.04, OR=6.21, 95% CL= 1.081–35.641), (p=0.049, OR=2.11, 95% CL=0.721–6.230) and (p=0.009, OR= 4.08, 95% CL= 1.427–11.685), respectively.

**Conclusion:**

Awareness of risk factors for heart attack appears to be poor, where most of the respondents recognized only one modifiable risk factor. According to these findings, programs and strategies to raise awareness of modifiable risk factors for HA are urgently needed to protect the lay public from HA.

## Introduction

Cardiovascular diseases (CVDs) are the leading causes of mortality globally, with an estimated 17.9 million deaths reported due to cardiovascular diseases in 2016, representing 31% of all global mortality.^1^ Ischemic heart disease is still the primary leading cause of mortality in Malaysia; in 2017, it accounted for 13.9% of all deaths in Malaysia.^2^ A heart attack is defined as a blockage in the arteries that supply the heart muscles with blood and oxygen, and it is typically characterized by chest pain, difficulty breathing, and pain in the neck and arms.^3^ Many risk factors contribute to heart attacks, such as hypertension, diabetes, obesity, hypercholesteremia, physical inactivity, unhealthy diet, smoking, stress, and atrial fibrillation.^1^ Risk factors for cardiovascular disease play an essential role in the development of ischemic heart disease.^4^ Therefore, knowledge and awareness of these risk factors for CVD play a significant role in preventing CVD and complications related to this disease.^5^

Several studies reported that a lack of awareness about disease risk factors and poor literacy rates in developing nations are associated with worse disease outcomes.^6^,^7^ Consequently, it was reported that CVD leads to high rates of hospital admissions and rising rates of mortality.^8–10^ One of the most common ways to reduce the CVD burden among the general public is to control modified risk factors (consumption of fatty foods, obesity, lack of working out, smoking, hypertension, diabetes, dyslipidemia, lack of exercise, stress).^4^ Knowledge of these modified risk factors can be used to introduce behavioral change strategies or targets through intervention programs.^11^,^12^ However, the paucity of education towards managing the risk factors for CVD can lead the public to fail to realize the signs and symptoms of CVD.^13^ The level of knowledge of the risk factors for heart attack in the general population is not given adequate attention.

Moreover, there are a limited number of studies conducted among patients and students in these areas.^14–16^ In a study involving South Asian family members, they were less likely to adopt lifestyle management and had lower awareness of a balanced diet or food content (fiber, sugar, salt).^17^,^18^ The assessment of the knowledge and awareness among the general public of CVD (heart attack) is thought to provide realistic insights that may be useful in helping advance public health policies toward reducing modifiable risk factors for CVD. The purpose of the current study is to determine the awareness of risk factors for heart attack among the general public in Kuantan, Pahang, Malaysia, and investigate the factors associated with the awareness of heart attack risk factors.

## Materials and Methods

### Study Design and Participants

This is a cross-sectional study conducted over two months, from May to July 2018, among the general public in Kuantan, Pahang, Malaysia. Through convenience sampling, the participants were recruited by approaching them at various local shopping centers and malls, mosques, churches, and eateries. Data were collected through face-to-face interviews with three trained staff who spoke Bahasa Melayu (local language) and English. Respondents who were presented with the eligibility requirements were invited to participate. The particular eligibility criteria were as follows: those aged 18–64 years old, healthy individuals. Those who were professionals in clinical and academic settings were excluded.

A total of 393 individuals were recruited. It was estimated using Roasoft^®^ (www.roasoft.com) online calculator to achieve 95% confidence interval level and accepted margin of error of 5% and considering a 50% response distribution. The sample size was obtained according to the population size obtained from the official portal of the Department of Statistics of Malaysia (https://www.dosm.gov.my/v1/index.php), ie, approximately 700,000 people at the time of the study.

### Research Instruments

This study was conducted using a validated standardized questionnaire that the research team had previously developed.^19^ The research questionnaire included three domains; the first one included social demographics such as (age, marital status, employment status, level of education, and monthly income). The second category included social habits and lifestyle. The last category included awareness of heart attack risk factors. Closed-ended questions were posed to the participants, and the potential responses were yes, no, or do not know. We described those who chose yes as knowing the risk factors and those who chose no or do not know as not being aware of risk factors for myocardial infarction.

### Ethical Requirements

Our study was approved by the International Islamic University Malaysia Research Ethics committee (IREC 2018–132). The study was conducted in accordance with the declaration of Helsinki. The study had been adequately explained to all participants, and they were given written informed consent. All individuals had also been guaranteed confidentiality, assured that they would remain entirely anonymous and understood that the information gathered would only be utilized for research purposes.

### Statistical Analysis

We used SPSS version 23 software (IBM Corp. Released 2015. IBM SPSS Statistics for Windows, Version 23.0. Armonk, NY: IBM Corp.) to analyze our data. We employed descriptive statistics in the form of frequencies and percentages of the social demographic variables. We used the chi-square test to obtain the association between social demographics and each risk factor for myocardial infarction. In addition, bivariate and multivariate logistic regressions were used to determine the factors associated with excellent knowledge for HA. Participants who identified all modifiable risk factors of heart attack was defined as “excellent awareness”. The following explanatory variables were included in the model dyslipidemia, gender, level of education, age, family history of HA, hypertension, diabetes, smoker, alcoholic, and those who heard of HA. Variables with significant p-value and p-value<0.25 were included in this model. The p-value <0.05 was considered statistically significant.

## Results

### Descriptive Statistics of Demographic Characteristics

Out of 393 individuals, 204 (51.9%) were males and 189 (48.1%) were females who residing in Kuantan, a city located on the eastern coast of Malaysia. The highest number of respondents was from the lowest age group (18–25), comprising (31%) 122 participants. Of these individuals, 53.7% were married. 65.1% of them had low education. Furthermore, of those surveyed, 71 were smokers, and 39 were alcoholics. Detailed information is shown in Table 1.

**Table 1:** Descriptive Statistics of Demographic Characteristics

Category	Subcategory	N	%
Gender	Male	204	51.9
	Female	189	48.1
Age (years)	18–25	122	31
	26–35	78	19.8
	36–45	65	16.5
	46–55	49	12.5
	56–64	79	20.1
Marital status	Single	174	44.3
	Married	211	53.7
	Divorced	5	1.3
	Widow	3	0.8
Education	Low education	256	65.1
	Good education	110	28
	High education	27	6.9
Employment status	Employed	265	67.4
	Unemployed	62	15.8
	Student	66	16.8
Race	Malay	213	54.2
	Chinese	119	30.3
	Indian	57	14.5
	Others	4	1.0
Monthly income	Less than 2000	269	68.4
	2000–3999	81	20.6
	4000–6000	42	10.7
	More than 6000	1	0.3
Medical history	Hypertension	67	17
	Diabetes	50	12.7
	Dyslipidemia	61	15.5
	Heart diseases	15	3.8
	Stroke	7	1.8
	Other diseases	4	1
Social habitus	Smoker	71	18.1
	Non-smoker	322	81.1
	Alcoholic	39	9.9
	Non-alcoholic	354	90.1

### Awareness of Individual Risk Factors for Heart Attack

The results related to the awareness of risk factors for heart attack and the relationship between sociodemographic and risk factors are shown in Table 2. The most common RFFHA recognized by the respondents was smoking (69.9%), followed by atrial fibrillation (57.7%), heart disease (54.1%), and obesity (53.8%). In addition, awareness of unhealthy diets and high cholesterol as RFFHAs comprised the same percentages (49.3%). However, less than 50% of the respondents were aware of stress, hypertension, alcohol, and family history of a heart attack as RFFHAs. Only 26.1% of the participants identified diabetes as a RFFHA, and 9.4% recognized a lack of exercise as a RFFHA. Generally, the result shows that respondents aged 36–45 years demonstrated more awareness of diabetes as a RFFHA than other age groups (*x*^2^=20.561, p=0.000). Furthermore, divorced respondents had more awareness of diabetes and stress as RFFHAs than others did (*x*^2^=22.071, p=0.000, and *x*^2^=8.767, p=0.033, respectively).

**Table 2:** Awareness of Each Individual’s Heart Attack Risk Factors

Percentage % of “Respondents Who Had Knowledge of RFFHA”												
Characteristics	Smoking	Obesity	Diabetic	Unhealthy Diet	Stress	Heart Disease	Hypertension	Alcohol Consumption	Genetic	Atrial Fibrillation	High Cholesterol	Lack of Exercise
Total	275 (69.9)	211 (53.8)	103 (26.1)	194 (49.3)	186 (47.4)	213 (54.2)	177 (44.8)	170 (43.2)	145 (36.9)	227 (57.7)	194 (49.3)	136 (9.4)
**Gender**												
Male	147 (72.1)	106 (52)	60 (29.4)	102 (50)	97 (47.5)	114 (55.9)	101 (49.5)	87 (42.6)	74 (36.3)	122 (59.8)	100 (49)	76 (37.3)
Female	128 (67.7)	105 (55.6)	43 (22.8)	92 (48.7)	89 (47.3)	99 (52.4)	76 (40.2)	83 (43.9)	71 (37.6)	105 (55.6)	94 (49.7)	60 (31.7)
**p-value**	0.34	0.47	0.13	0.79	0.92	0.48	0.06	0.80	0.79	0.39	0.88	0.10
**Age**												
18–25	83 (67.8)	70 (57.4)	17 (14)	58 (47.9)	49 (40.5)	62 (51.2)	51 (41.1)	53 (43.4)	40 (32.2)	70 (57.9)	61 (49.6)	40 (32.8)
26–35	52 (66.7)	42 (53.8)	19 (24.4)	35 (44.9)	42 (53.8)	45 (57.7)	30 (38.5)	38 (48.7)	24 (30.8)	50 (64.1)	41 (52.6)	29 (37.2)
36–45	53 (81.5)	32 (49.2)	26 (40)	34 (52.3)	32 (49.2)	36 (55.4)	37 (56.9)	34 (52.3)	31 (47.7)	37 (56.9)	33 (50.8)	24 (36.9)
46–55	34 (69.4)	28 (57.1)	12 (24.5)	27 (55.1)	25 (51)	25 (51)	25 (51)	19 (38.8)	20 (40.8)	25 (51)	24 (49)	20 (40.8)
56–64	53 (67.1)	39 (49.4)	29 (36.7)	40 (50.6)	38 (48.1)	45 (57)	34 (43)	26 (32.9)	30 (38)	45 (57)	35 (44.3)	23 (29.1)
**p-value**	0.35	0.73	0.000*	0.75	0.43	0.78	0.21	0.14	0.17	0.60	0.81	0.99
**Marital Status**												
Single	116 (66.7)	96 (55.2)	26 (14.9)	78 (44.8)	71 (40.8)	92 (52.9)	70 (40.2)	76 (43.7)	54 (31)	100 (57.5)	88 (50.6)	55 (31.6)
Married	154 (73)	112 (53.1)	73 (34.6)	112 (53.1)	112 (53.1)	117 (55.5)	103 (48.8)	93 (44.1)	88 (41.7)	121 (57.3)	105 (49.8)	78 (37.0)
Divorced	3 (60)	2 (40)	3 (60)	3 (60)	3 (60)	4 (80)	2 (40)	0 (0.0)	2 (40)	4 (80)	1 (20)	2 (40)
Widow	2 (66.7)	1 (33.3)	1 (33.3)	1 (33.3)	0 (0.0)	0 (0.0)	2 (66.7)	1 (33.3)	1 (33.3)	2 (66.7)	0 (0.0)	1 (33.3)
**p-value**	0.55	0.78	0.000*	0.37	0.03*	0.16	0.32	0.26	0.19	0.76	0.19	0.77
**Race**												
Malay	162 (76.1)	125 (58.7)	54 (25.4)	119 (55.9)	109 (51.2)	116 (54.5)	91 (42.7)	91 (42.7)	81 (38)	134 (62.9)	113 (53.1)	78 (36.6)
Chinese	76 (63.9)	64 (53.8)	40 (33.6)	54 (45.4)	56 (47.1)	70 (58.8)	64 (53.8)	58 (48.7)	54 (45.4)	64 (53.8)	59 (49.6)	47 (39.5)
Indian	34 (59.6)	21 (36.8)	9 (15.8)	20 (35.1)	19 (33.3)	25 (43.9)	22 (38.6)	19 (33.3)	10 (17.5)	27 (47.4)	21 (36.8)	9 (15.8)
Others	3 (75)	1 (25)	0 (0.0)	1 (25)	2 (50)	2 (50)	0 (0.0)	2 (50)	0 (0.0)	2 (50)	1 (25)	2 (50)
**p-value**	0.03*	0.01*	0.04*	0.01*	0.12	0.31	0.03*	0.27	0.00*	0.12	0.12	0.00*
**Education**												
Lower education	171 (66.8)	130 (50.8)	78 (30.5)	120 (46.9)	121 (47.3)	129 (50.4)	112 (43.8)	100 (39.1)	97 (37.9)	134 (52.3)	116 (45.3)	88 (34.4)
Good education	83 (75.5)	60 (54.5)	20 (18.2)	58 (52.7)	51 (46.4)	62 (56.4)	50 (45.5)	53 (48.2)	39 (35.5)	75 (68.2)	63 (57.3)	38 (34.5)
High education	21 (77.8)	21 (77.8)	5 (18.5)	16 (59.3)	14 (51.9)	22 (81.5)	15 (55.6)	17 (63)	9 (33.3)	18 (66.7)	15 (55.6)	10 (37)
**p-value**	0.16	0.02*	0.03*	0.33	0.87	0.00*	0.50*	0.02*	0.83	0.01*	0.08*	0.96
**Employment**												
Employed	186 (70.2)	138 (52.1)	73 (27.5)	123 (46.4)	134 (50.6)	143 (54)	120 (45.3)	116 (43.8)	95 (35.8)	158 (59.6)	130 (49.1)	91 (34.3)
Unemployed	43 (69.4)	35 (56.5)	20 (32.3)	33 (53.2)	28 (45.2)	31 (50)	29 (46.8)	25 (40.3)	26 (41.9)	30 (48.4)	27 (43.5)	21 (33.9)
Student	46 (69.7)	38 (57.6)	10 (15.2)	38 (57.6)	24 (36.4)	39 (59.1)	28 (42.4)	29 (43.9)	24 (36.4)	39 (59.1)	37 (56.1)	24 (36.4)
**p-value**	0.99	0.64	0.05	0.27	0.11	0.58	0.87	0.87	0.66	0.26	0.36	0.94
**Income**												
Less than 2000	181 (67.3)	144 (53.5)	67 (24.9)	128 (47.6)	115 (42.8)	140 (52)	114 (42.4)	103 (38.3)	94 (34.9)	143 (53.2)	119 (44.2)	88 (32.7)
2000–3999	59 (72.8)	42 (51.9)	24 (29.6)	43 (53.1)	45 (55.6)	44 (54.3)	37 (45.7)	43 (53.1)	32 (39.5)	52 (64.2)	47 (58)	33 (40.7)
4000–6000	34 (81)	24 (57.1)	12 (28.6)	23 (54.8)	25 (59.5)	28 (66.7)	26 (61.9)	23 (54.8)	19 (45.2)	31 (73.8)	28 (66.7)	15 (35.7)
More than 6000	1 (100)	1 (100)	0 (0.0)	0 (0.0)	1 (100)	1 (100)	0 (0.0)	1 (100)	0 (0.0)	1 (100)	0 (0.0)	0 (0.0)
**p-value**	0.25	0.75	0.75	0.52	0.04*	0.26	0.09	0.02*	0.47	0.03*	0.01*	0.27
**Heard about heart attack**												
Yes	239 (71.6)	193 (57.8)	96 (28.7)	173 (51.8)	169 (50.6)	191 (57.2)	155 (46.4)	152 (45.5)	138 (41.3)	202 (60.5)	174 (52.1)	130 (38.9)
No	36 (61)	18 (30.5)	7 (11.9)	21 (35.6)	17 (28.8)	22 (37.3)	22 (37.3)	18 (30.5)	7 (11.9)	25 (42.4)	20 (33.9)	6 (10.2)
**p-value**	0.103	0.000*	0.00*	0.02*	0.00*	0.00*	0.19	0.03*	0.00*	0.00*	0.01*	0.00*
**Hypertension**												
Yes	47 (70.1)	40 (59.7)	17 (25.4)	35 (52.2)	30 (44.8)	39 (58.2)	32 (47.8)	33 (49.3)	26 (38.8)	38 (56.7)	33 (49.3)	27 (40.3)
No	228 (69.9)	171 (52.5)	86 (26.4)	159 (48.8)	156 (47.9)	174 (53.4)	145 (44.5)	137 (42)	119 (36.5)	189 (58)	161 (49.4)	109 (33.4)
**p-value**	0.97	0.27	0.86	0.60	0.64	0.46	0.62	0.27	0.72	0.84	0.98	0.28
**Diabetics**												
Yes	32 (64)	23 (46)	12 (24)	29 (58)	24 (48)	30 (60)	23 (46)	20 (40)	18 (36)	32 (64)	24 (48)	20 (40)
No	243 (70.8)	188 (54.8)	91 (26.5)	165 (48.1)	162 (47.2)	183 (53.4)	154 (44.9)	150 (43.7)	127 (37)	195 (56.9)	170 (49.6)	116 (33.8)
**p-value**	0.32	0.24	0.70	0.19	0.91	0.37	0.88	0.61	0.88	0.33	0.83	0.39
**Dyslipidemia**												
Yes	43 (70.5)	34 (55.7)	26 (42.6)	34 (55.7)	28 (45.9)	35 (57.4)	29 (47.5)	30 (49.2)	27 (44.3)	39 (63.9)	35 (57.4)	32 (52.5)
No	232 (69.9)	177 (53.3)	77 (23.2)	160 (48.2)	158 (47.6)	178 (53.6)	148 (44.6)	140 (42.2)	118 (35.5)	188 (56.6)	159 (47.9)	104 (31.3)
**p-value**	0.92	0.72	0.00*	0.27	0.80	0.58	0.66	0.31	0.19	0.28	0.17	0.00*
**History of HA among relatives, acquaintances, or neighbors**												
Yes	157 (71.7)	131 (59.8)	59 (26.9)	123 (56.2)	110 (50.2)	125 (57.1)	102 (46.6)	98 (44.7)	86 (39.3)	125 (57.1)	113 (51.6)	87 (39.7)
No	118 (67.8)	80 (46)	44 (25.3)	71 (40.8)	76 (43.7)	88 (50.6)	75 (43.1)	72 (41.4)	59 (33.9)	102 (58.6)	81 (46.6)	49 (28.2)
**p-value**	0.40	0.00*	0.71	0.00*	0.19	0.19	0.49	0.50	0.27	0.75	0.32	0.01*
**Being aware that heart attack requires prompt treatment**												
Yes	255 (72.6)	200 (57)	98 (27.9)	182 (51.9)	172 (49)	195 (55.6)	162 (46.2)	157 (44.7)	132 (37.6)	207 (59)	178 (50.7)	128 (36.5)
Sometimes	11 (47.8)	8 (34.8)	4 (17.4)	7 (30.4)	8 (34)	9 (39.1)	8 (34.8)	9 (39.1)	9 (39.1)	10 (43.5)	10 (43.5)	3 (13)
No	9 (47.4)	3 (15.8)	1 (5.3)	5 (26.3)	6 (31.6)	9 (47.4)	7 (36.8)	4 (21.1)	4 (21.1)	10 (52.6)	6 (31.6)	5 (26.3)
**p-value**	0.00*	0.00*	0.05	0.01*	0.15	0.25	0.43	0.11	0.33	0.31	0.22	0.05
**Have you received any information related to public service announcements, promotional materials, or educational leaflets?**												
Yes	209 (72.1)	168 (57.9)	87 (30)	153 (52.8)	140 (48.3)	164 (56.6)	138 (47.6)	136 (46.9)	118 (40.7)	178 (61.4)	156 (53.8)	112 (38.6)
No	66 (64.1)	43 (41.7)	16 (15.5)	41 (39.8)	46 (44.7)	49 (47.6)	39 (37.9)	34 (33)	27 (26.2)	49 (47.6)	38 (36.9)	24 (23.3)
p-value	0.12	0.00*	0.00*	0.02*	0.52	0.11	0.08	0.01*	0.00*	0.01*	0.00*	0.00*

**Notes:** Atrial fibrillation (the phrase “Irregular heart pulse” was used in the survey). Notice: *Statistical significantly at *P*-value <0.05.

Malay respondents showed greater awareness of smoking (*x*^2^=8.782, p=0.032), obesity (*x*^2^ =9.944, p=0.019), and unhealthy diet (*x*^2^ =9.934, p=0.019) than their counterparts did. Chinese participants, however, had more awareness of diabetes (*x*^2^ =8.054, p=0.045), hypertension (*x*^2^=8.348, p=0.039), genetics (*x*^2^ =15.263, p=0.002), and lack of exercise (*x*^2^ =10.948, p=0.012) as RFFHAs than other races did. In addition, high education respondents (master’s and PhD level) demonstrated better awareness of obesity, heart disease, alcohol consumption, atrial fibrillation, and diabetic than did others who had good education and low education respondents (*x*^2^ =7.186, p=0.028; *x*^2^ =6.875, p=0.032; *x*^2^ =9.774, p=0.008; *x*^2^ =8.831, p=0.012; and *x*^2^ =7.175, p=0.028, respectively). Moreover, respondents with high income were more likely to identify stress, alcohol consumption, atrial fibrillation, and dyslipidemia as RFFHAs than those with less income were (*x*^2^ =8.058, p=0.045; *x*^2^ =9.445, p=0.024; *x*^2^ =8.852, p=0.031; and *x*^2^ =11.235, p=0.011, respectively). Respondents who had heard about HA and heart disease were more likely to identify all RFFHAs than were others who had not.

Furthermore, participants diagnosed with dyslipidemia were more likely to recognize diabetes and lack exercise as RFFHAs than others without dyslipidemia (*x*^2^=8158, p=0.002 *x*^2^=7986, p=0.001, respectively). Regarding the family history of HA among the participants, those with a family history of HA among relatives, acquaintances, and neighbors demonstrated greater awareness of RFFHAs, such as obesity, unhealthy diet, and lack of exercise, than were those without a family history of a heart attack. Additionally, respondents who had received any information related to HA through public service announcements, promotional materials, and educational leaflets showed more awareness of RFFHAs than did others who did not receive this information. Finally, respondents who were aware that HA requires urgent medication had a better awareness of smoking (*x*^2^=11.159, p=0.004), obesity (*x*^2^=15.772, p=0.000), and unhealthy diet (*x*^2^=8.183, p=0.017) as RFFHAs than did others who were unaware that heart attack and stroke require quick treatment. Detailed information is shown in Table 2.

### Awareness of Modifiable Risk Factors for Heart Attack Using the Number of Risk Factors

Most of the respondents (90.6%) identified at least one modifiable risk factor for heart attack (MRFFHA), while 9.8% did not identify any MRFFHAs. However, 22 respondents (5.6%) identified all MRFFHAs. There were no significant differences between males and females regarding the awareness of all MRFFHAs (U=19,195, p=0.854). Participants aged 46–55 years old were more likely to identify eight MRFFHAs than those aged 18–45 years old (*x*^2^=11.856, p=0.018). Similarly, married respondents showed more awareness of eight MRFFHAs than were others who were single (*x*^2^=9.769, p=0.02). Likewise, Malay respondents were more likely to identify four MRFFHAs than were respondents of other races (*x*^2^=7.139, p=0.028). However, Chinese respondents had a greater awareness of eight MRFFHAs than respondents of other races (*x*^2^=6.632, p=0.036). Furthermore, participants with higher education (postgraduate) were more likely to know five MRFFHAs than those with low education and good education (*x*^2^=5.739, p=0.047). Furthermore, participants with high monthly income demonstrated better awareness of two MRFFHAs than were others with low income (*x*^2^=8.714, p=0.033). Moreover, participants who heard about HA and heart disease had a great awareness of eight MRFFHAs (*x*^2^=8820, p=0.033). Similarly, respondents who received information about HA through public advertisements or social media showed better awareness of all nine MRFFHAs than those who did not receive any information (*x*^2^=13,802, p=0.004). However, participants who were aware that HA requires quick treatment were more likely to identify four MRFFHAs than others unaware that HA requires urgent treatment (*x*^2^=13.824, p=0.001). In addition, respondents with a family history of heart disease among relatives or neighbors demonstrated significant and better awareness of all MRFFHAs than did others without this condition (*x*^2^=18,121, p=0.036). Moreover, diabetic and hypercholesterolemic respondents were more aware of all MRFFHAs than were others without diabetes and dyslipidemia (*x*^2^=7749.5, p=0.006 and *x*^2^=9028.5, p=0.001, respectively).

On the other hand, there was no significant difference between hypertensive respondents and employment status and awareness of each MRFFHA. Additionally, there were no significant differences between awareness of MRFFHAs and gender, age, marital status, race, education, income, employment status, hypertension, awareness that heart attack requires quick medication, or information on HA through a public service announcement or social media (see Table 3).

**Table 3:** Awareness of Modifiable Risk Factors for Heart Attack by the Number of Risk Factors

Percentage % of “Respondents Who Had Knowledge According to the Number of RFFHA”									
Characteristics	1≥	2≥	3≥	4≥	5≥	6≥	7≥	8≥	^A^Excellent Awareness
Total	356 (90.6%)	316 (80.4%)	262 (66.7%)	222 (56.5%)	175 (44.5%)	142 (36.1%)	96 (24.4%)	55 (14%)	22 (5.6%)
**Gender**									
Male	186 (91.2)	160 (78.4)	141 (69.1)	118 (57.8)	98 (48)	77 (37.7)	53 (26)	32 (15.7)	11 (5.4)
Female	170 (89.9)	156 (82.5)	121 (64)	104 (55)	77 (40.7)	65 (34.4)	43 (22.8)	23 (12.2)	11 (5.8)
p-value	0.67	0.30	0.28	0.57	0.14	0.48	0.45	0.31	0.85
**Age**									
18–25	113 (92.6)	99 (81.1)	82 (67.2)	67 (54.9)	50 (41)	38 (31.1)	22 (18)	9 (7.4)	2 (1.6)
26–35	73 (93.6)	66 (84.6)	52 (66.7)	45 (57.7)	34 (43.6)	26 (33.3)	17 (21.8)	9 (11.5)	6 (7.7)
36–45	61 (93.8)	55 (84.6)	48 (73.8)	43 (66.2)	35 (53.8)	30 (46.2)	19 (29.2)	9 (13.8)	5 (7.7)
46–55	45 (91.8)	39 (79.6)	33 (67.3)	27 (55.1)	21 (42.9)	20 (40.8)	15 (30.6)	12 (24.5)	2 (4.1)
56–64	64 (81)	57 (72.2)	47 (59.5)	40 (50.6)	35 (44.3)	28 (35.4)	23 (29.1)	16 (20.3)	7 (8.9)
p-value	0.02*	0.27	0.49	0.43	0.55	0.29	0.21	0.01*	0.15
**Marital status**									
Single	159 (91.4)	139 (79.9)	113 (64.9)	93 (53.4)	69 (39.7)	52 (29.9)	31 (17.8)	15 (8.6)	5 (2.9)
Married	192 (91)	172 (81.5)	145 (68.7)	125 (59.2)	102 (48.3)	86 (40.8)	63 (29.9)	40 (19)	17 (8.1)
Divorced	3 (60)	3 (60)	3 (60)	3 (60)	3 (60)	3 (60)	1 (20)	0 (0.0)	0 (0.0)
Widow	2 (66.7)	2 (66.7)	1 (33.3)	1 (33.3)	1 (33.3)	1 (33.3)	1 (33.3)	0 (0.0)	0 (0.0)
p-value	0.05	0.59	0.52	0.57	0.31	0.10	0.05	0.02*	0.14
**Race**									
Malay	202 (94.8)	173 (82.2)	151 (70.9)	127 (59.6)	101 (47.4)	84 (39.4)	58 (27.2)	32 (15)	12 (5.6)
Chinese	103 (86.6)	96 (80.7)	80 (67.2)	70 (58.8)	57 (47.9)	49 (41.2)	33 (27.7)	21 (17.6)	9 (7.6)
Indian	48 (84.2)	42 (73.7)	29 (50.9)	23 (40.4)	16 (28.1)	8 (14)	5 (8.8)	2 (3.5)	1 (1.8)
Others	3 (75)	3 (75)	2 (50)	2 (50)	1 (25)	1 (25)	0 (0.0)	0 (0.0)	0 (0.0)
p-value	0.01*	0.54	0.03*	0.06	0.04*	0.00*	0.01*	0.06	0.44
**Income**									
Less than 2000	239 (88.8)	206 (76.6)	170 (63.2)	143 (53.2)	111 (41.3)	88 (32.7)	58 (21.6)	32 (11.9)	12 (4.5)
2000–3999	74 (91.4)	70 (86.4)	58 (71.6)	52 (64.2)	41 (50.6)	34 (42)	22 (27.2)	15 (18.5)	7 (8.6)
4000–6000	42 (100)	39 (92.9)	33 (78.6)	26 (61.9)	23 (54.8)	20 (47.6)	16 (38.1)	8 (19)	3 (7.1)
More than 6000	1 (100)	1 (100)	1 (100)	1 (100)	0 (0.0)	0 (0.0)	0 (0.0)	0 (0.0)	0 (0.0)
p-value	0.14	0.03*	0.13	0.21	0.17	0.13	0.10	0.33	0.50
**Heard about heart attack**									
Yes	311 (93.1)	277 (82.9)	237 (71)	201 (60.2)	160 (47.9)	131 (39.2)	91 (27.2)	52 (15.6)	21 (6.3)
No	45 (76.3)	39 (66.1)	25 (42.4)	21 (35.6)	15 (25.4)	11 (18.6)	5 (8.5)	3 (5.1)	1 (1.7)
p-value	0.00*	0.00*	0.00*	0.00*	0.00*	0.00*	0.00*	0.03*	0.15
**Employment**									
Employed	237 (89.4)	210 (79.2)	170 (64.2)	147 (55.5)	118 (44.5)	100 (37.7)	70 (26.4)	41 (15.5)	18 (6.8)
Unemployed	54 (87.1)	48 (77.4)	42 (67.7)	36 (58.1)	29 (46.8)	21 (33.9)	16 (25.8)	11 (17.7)	4 (6.5)
Student	65 (98.5)	58 (87.9)	50 (75.8)	39 (59.1)	28 (42.4)	21 (31.8)	10 (15.2)	3 (4.5)	0 (0.0)
p-value	0.04*	0.23	0.19	0.83	0.88	0.61	0.15	0.04*	0.09
**Education**									
Lower education	222 (86.7)	195 (76.2)	159 (62.1)	142 (55.5)	110 (43)	90 (35.2)	60 (23.4)	39 (15.2)	19 (7.4)
Good education	107 (97.3)	96 (87.3)	83 (75.5)	61 (55.5)	47 (42.7)	40 (36.4)	28 (25.5)	11 (10)	3 (2.7)
High education	27 (100)	25 (92.6)	20 (74.1)	19 (70.4)	18 (66.7)	12 (44.4)	8 (29.6)	5 (18.5)	0 (0.0)
p-value	0.00*	0.01*	0.03*	0.32	0.05*	0.63	0.74	0.32	0.08
**Hypertension**									
Yes	61 (91)	55 (82.1)	43 (64.2)	40 (59.7)	34 (50.7)	25 (37.3)	19 (28.4)	11 (16.4)	6 (9)
No	295 (90.5)	261 (80.1)	219 (67.2)	182 (55.8)	141 (43.3)	117 (35.9)	77 (23.6)	44 (13.5)	16 (4.9)
p-value	0.88	0.70	0.63	0.56	0.26	0.82	0.41	0.53	0.18
**Hypercholesterolemia**									
Yes	58 (95.1)	53 (86.9)	44 (72.1)	39 (63.9)	31 (50.8)	25 (41)	19 (31.1)	13 (21.3)	9 (14.8)
No	298 (89.8)	263 (79.2)	218 (65.7)	183 (55.1)	144 (43.4)	117 (35.2)	77 (23.2)	42 (12.7)	13 (3.9)
p-value	0.19	0.16	0.32	0.20	0.28	0.39	0.18	0.07	0.00*
**History of HA among relatives, acquaintances, neighbors**									
Yes	201 (91.8)	186 (84.9)	155 (70.8)	134 (61.2)	106 (48.4)	86 (39.3)	60 (27.4)	35 (16)	17 (7.8)
No	155 (89.1)	130 (74.7)	107 (61.5)	88 (50.6)	69 (39.7)	56 (32.2)	36 (20.7)	20 (11.5)	5 (2.9)
p-value	0.36	0.01*	0.05	0.03*	0.08	0.14	0.12	0.20	0.03*
**Being aware that heart attack requires prompt treatment**									
Yes	328 (93.4)	294 (83.8)	245 (69.8)	209 (59.5)	163 (46.4)	133 (37.9)	89 (25.4)	50 (14.2)	21 (6)
Sometimes	15 (65.2)	12 (52.2)	9 (39.1)	9 (39.1)	8 (34.8)	6 (26.1)	5 (21.7)	4 (17.4)	0 (0.0)
No	13 (68.4)	10 (52.6)	8 (42.1)	4 (21.1)	4 (21.1)	3 (15.8)	2 (10.5)	1 (5.3)	1 (5.3)
p-value	0.00*	0.00*	0.00*	0.00*	0.06	0.08	0.32	0.48	0.48
**Have you received any information related to public service announcements, promotional materials, or educational leaflets?**									
Yes	268 (92.4)	242 (83.4)	204 (70.3)	173 (59.7)	141 (48.6)	117 (40.3)	82 (28.3)	50 (17.2)	22 (7.6)
No	88 (85.4)	74 (71.8)	58 (56.3)	49 (47.6)	34 (33)	25 (24.3)	14 (13.6)	5 (4.9)	0 (0.0)
p-value	0.03*	0.01*	0.00*	0.03*	0.00*	0.00*	0.00*	0.00*	0.00*

**Notes:** *Statistically significant at P-value <0.05. (1≥), respondents know at least one RFOHA, (2≥) respondents know at least 2 RFOHA, (3≥) respondents know 3 RFOHA, (4≥) respondents know 4 RFOHA, (5≥) respondents know 5 RFOHA, (6≥) respondents know 6 RFOHA, (7≥) respondents know 7 RFOHA, (8≥) respondents know 8 RFOHA, ^A^(Excellent awareness, knows all modifiable risk factors for heart attack).

### Factors Independently Related to an Excellent Awareness of Risk Factors for Heart Attack

Bivariate logistic regression illustrated the factors related with an excellent awareness for RFFHA. Respondents with age 55–64, those with less university educated, with family history for HA, dyslipidaemia, consumed alcohol, and with currently diabetic showed greater knowledge related with an excellent awareness (p= 0.03, OR=5.83, 95% CL=1.180–28.846), (p=0.04, OR=3.58,95% CL=1.040–12.324), (p=0.04, OR= 2.84, 95% CL=1.028–7.872), (p=0.04, OR= 2.91, 95% CL=1.012–8.395) and (p=0.009, OR= 3.56, 95% CL=1.374–9.221), respectively (Table 4).

**Table 4:** Factors Associated with Excellent Awareness According to Risk Factors for Heart Attack

Parameter	Sample Logistic Regression		Multivariate Logistic Regression	
	O.R. (95% CI)	p-value	O.R. (95% CI)	p-value
**Age**				
18–25	Ref		Ref	
26–35	5.00(0.983–25.437)	0.05*	4.90(0.882–27.281)	0.06
36–45	5.00(0.942–26.530)	0.05*	5.09(0.854–30.429)	0.07
46–55	2.55(0.349–18.655)	0.35	3.25(0.388–27.229)	0.27
55–64	5.83(1.180–28.846)	0.03*	6.21(1.081–35.692)	0.04*
**Education**				
Below university	3.58(1.040–12.324)	0.04*	2.21(0.601–8.141)	0.23
University	Ref		Ref	
**Gender**				
Male	0.92(0.390–2.180)	0.85	0.75(0.290–1.947)	0.55
Female	Ref		Ref	
**Family history of HA**				
Yes	2.84(1.028–7.872)	0.04*	2.11(0.721–6.230)	0.049*
No	Ref		Ref	
**Heard about HA**				
Yes	3.89(0.513–29.498)	0.18	4.05(0.477–34.4333)	0.20
No	Ref		Ref	
**Smoker**				
Yes	0.70(0.202–2.445)	0.58	0.39(0.098–1.621)	0.19
No	Ref		Ref	
**Hypertension**				
**Yes**	1.90(0.717–5.066)	0.19	1.27(0.415–3.921)	0.10
**No**	Ref		Ref	
**Dyslipidaemia**				
Yes	4.24(1.728–10.436)	0.00*	4.08(1.427–11.685)	0.00*
No	Ref		Ref	
**Alcoholic**				
Yes	2.91(1.012–8.395)	0.04*	2.71(0.804–9.175)	0.10
No	Ref		Ref	
**Diabetic**				
Yes	3.56(1.374–9.221)	0.009*	2.36(0.663–8.825)	0.20
No	Ref		Ref	

**Note:** *Statistically significant at <0.05.

**Abbreviations:** OR, odds ratio; Cl, confidence interval.

As demonstrated in Table 4, factors independently related to an excellent awareness in the multivariate logistic regression model were age, those who had dyslipidemia and individuals with family history of HA. The knowledge about an excellent awareness of RFFHA was greater among those aged 55–64 years old than other age groups (p=0.04, OR=6.21, 95% CL= 1.081–35.641). Moreover, individuals with dyslipidemia were 4.08 times more likely to identify an excellent awareness of RFFHA (p=0.009, OR= 4.08, 95% CL= 1.427–11.685). In addition, participants with family history of HA were 2.11 times more likely to recognize the excellent awareness of RFFHA (p=0.049, OR=2.11, 95% CL=0.721–6.230).

## Discussion

This study aimed to examine the general public’s awareness of risk factors for heart attack and investigate the factors associated with awareness of risk factors for heart attack. There was poor awareness among them. Most of the respondents recognized only one modifiable risk factor. According to these findings, programs and strategies to raise awareness of modifiable risk factors for HA are urgently needed to protect the lay public from HA.

Knowledge and understanding of risk factors for heart attack are essential in enabling people to change their behavior and attitudes towards CHD prevention. Early prevention and changes in sedentary lifestyle behaviors remain the best approaches.^20^ The control of modifiable risk factors for heart attack is essential to prevent cardiovascular diseases. In this study, the awareness of risk factors for heart attack was found to be poor. However, the results are comparable with previously conducted studies in Asia.^21^,^22^ If the populace, particularly the youth, has a good perception of the risk factors for CVD, they will be capable of adopting primary preventive measures earlier in their lives.^23^ It could be assumed that smoking is the principal cause of nearly one-third of the deaths by CVDs and was recognized as a risk factor for heart attack by the highest proportion of respondents in the US.^24^ Similarly, it was the most significant risk factor identified by the participants in this research.

Furthermore, a lower number of respondents did not recognize any risk factors for heart attack in the current study. However, most of the participants were aware of the risks associated with smoking, obesity, and unhealthy diet. The findings were comparable to those of other studies in Malaysia, Kuwait, Thailand, Jordan, and Canada.^16^,^25–29^ Similarly, a study on the northeastern coast of Malaysia reported that the major risk factors for CVDs were smoking, hypertension, obesity, stress, and diabetes.^30^ The highest proportion related to the awareness of smoking as a risk factor of HA was discovered in the current research, as well as in other studies, local and international, because smoking is a well-known major risk factor for CVD. Many smoking cessation campaigns conducted both locally and globally influenced the lay public’s awareness regarding the negative health impact of smoking.^31^,^32^ Similar findings have been reported in other studies.^11^,^21^,^33^ This may indicate that lack of knowledge regarding heart attack is a global concern. The current research results are consistent with previous studies, highlighting the urgent need to develop tailor-made strategies to improvise awareness of cardiovascular diseases and their risk factors.^21^,^33–36^ However, in this study, most of the respondents were not aware of diabetes as a RFFHA, although diabetes is a leading risk factor for heart attack and stroke.^37^ These findings were supported by the studies done in Korea, Greece, Emirates, and Jordan, which demonstrated that diabetes accounted for the lowest percentage of RFFHAs.^23^,^28^,^38^,^39^ The findings of our study highlighted the improvement of general public awareness regarding risk factors for HA with more emphasis being laid on diabetes, which is highly prevalent among Malaysians. The healthcare professional messages must promote positive health behaviors and communicate to lay public that awareness and action towards risk factors for HA are interrelated with decreased morbidity and mortality. Healthcare professionals should be assured to impart education to lay public in general and patients in particular.

A heart attack is the most eminent cause of death and morbidity, while risk factors for HA should be well known to the general public to avoid heart attack-driven death.^1^,^2^ In addition, less than half of the respondents in the present study recognized hypertension, dyslipidemia, stress, family history (genetics), alcohol consumption, and physical inactivity as risk factors for heart attack. This finding is consistent with those of studies in Jordan and Canada.^28^,^29^ In contrast, studies in Thailand, Emirates, and Kuwait reported a higher level of knowledge of RFFHAs, such as hypertension (91.3%), dyslipidemia (95.9%), stress (88%), genetics (96%), alcohol consumption (70%) and sedentary lifestyle (93%).^23^,^25^,^27^ The awareness of RFFHAs in Kuwait might reflect the intensive mass media campaigns about risk factors for CVD.^25^ At the same time, in Emirates, the government had invested significantly in many health promotional campaigns to increase community health and knowledge of health and lifestyles.^31^ However, in Malaysia, especially in Kuantan and although several health education initiatives by the Ministry of health, there is still a need for well-structured awareness campaigns on cardiovascular disease (heart attack and stroke).

Concerning the number of RFFHAs, only a few respondents identified at least one or two modifiable risk factors in the current research. At the same time, the majority were not aware of all modifiable RFFHAs. These findings are in line with those of studies in Pakistan, Kuwait, and Emirates.^21–23^,^25^ As reported earlier, in this research, most of the participants were not aware of RFFHAs, such as hypertension, dyslipidemia, stress, and lack of exercise. This reflects a lack of awareness among lay people with lower incomes and high school education. The lack of intensive mass media campaigns on the risk factors and symptoms of a heart attack may also be responsible. In addition, there was an association in the current research between a high level of education and monthly income and those at high risk of HA, those who heard about HA, those who received HA-related information, and those who were well aware of RFFHAs. These findings are similar to those of other studies in Kuwait, Emirates, and Pakistan.^22^,^23^,^25^

The current study discovered that respondents who stated having family history of HA had great knowledge of RFFHA compared to respondents who did not state having a family history of HA. These key findings are consistent with the American, Northern Irish and Jordanian studies which showed that participants who had a family history of CVD paid attention to their lifestyle than those who did not have a family history of CVD.^28^,^40^ Individuals with aged 55–64, family history and those with diabetes were found to be significantly related with an excellent awareness of RFFHA. This finding is supported with studies in Kuwait that mentioned that the high knowledge about CVD in middle age and elderly age might be associated with their less working hours and they have more time to use mass media like radio, television, and newspapers compared to the young people.^25^,^41^ However, our study demonstrated that no significant difference in RFFHA between who were smokers and did not, which is similar to a study conducted in Northern Irish.^33^

## Limitations

Due to the financial constrain and time, the study was focused only in one city, and convenience sampling techniques were used. That limits finding generalizability. However, the social-demographic, mean salary, education, and culture were similar with other parts of the country. Another limitation is the type of study design, ie, an observational design. The nature of the study and the lack of control group which could not identify some potential confounders. Further studies are recommended, with a large sample size covering more towns and random samples with educational intervention.

## Study Implications

As mentioned earlier, strategies to raise awareness of modifiable risk factors for HA are urgently needed to protect the lay public from HA. It may help to reduce the public’s exposure to modifiable risk factors. It then can contribute to the prevention and control strategies. The state health authorities can design the programs and strategies to enhance the knowledge and coping skills toward the risk factors. In collaboration with university hospitals, the authorities can establish health promotion programs and campaigns through various media (digital and non-digital) to improve knowledge and increase awareness. Engaging healthcare profession students in universities, especially in the senior years, should be well planned. Some public hospitals do home care, which can also be used to reach the public and provide health education. Additionally, primary healthcare providers such as private general practitioners and community pharmacists should be encouraged. Many people visit primary healthcare practitioners for healthcare services. In short, various means are cost-effective can be implemented to increase awareness in the community.

## Conclusions

The awareness of modifiable risk factors for heart attack among Kuantan, Malaysia, was poor among the lay public. Most of them recognized only one modifiable risk factor for a heart attack. Furthermore, a lower number of respondents identified all modifiable risk factors for HA. Participants aged 55–64, those with family history of heart attack, and individuals with dyslipidemia were factors independently related to excellent awareness. According to these findings, programs and strategies to raise awareness of modifiable risk factors for HA are urgently needed to protect the lay public from HA.

## Abbreviations

CVDs, cardiovascular diseases; CHD, coronary heart disease; HA, heart attack; RFFHA, risk factors for heart attack; MRFFHA, modifiable risk factors for heart attack; OR, odds ratio; CL, confidence interval.
